# Estimation of endogenous protein and amino acid ileal losses in weaned piglets by regression analysis using diets with graded levels of casein

**DOI:** 10.1186/2049-1891-4-36

**Published:** 2013-09-22

**Authors:** Tércia Cesária Reis de Souza, Araceli Aguilera Barreyro, Gerardo Mariscal-Landín

**Affiliations:** 1 Facultad de Ciencias Naturales, Universidad Autónoma de Querétaro, Santiago de Querétaro, Av. De las Ciencias s/n Juriquilla 76000, Querétaro, Querétaro, México; 2 Centro Nacional de Investigación en Fisiología Animal, Instituto Nacional de Investigaciones Forestales Agrícolas y Pecuarias, Km 1, Carretera a Colón, Ajuchitlán Colón Querétaro, 76280, México

**Keywords:** Amino acid loss, Ileal digestibility, Protein loss, Weaned piglets

## Abstract

**Background:**

Many studies have investigated endogenous loss of proteins and amino acids (AAs) at the ileal level in growing pigs. However, only a few studies have researched this subject in piglets. Knowledge regarding AA ileal digestibility in piglets would be helpful during the formulation of diets for weaning piglets, rather than just using coefficients obtained in growing pigs. Therefore, in this study, we sought to estimate endogenous protein and AA ileal losses in piglets. Furthermore, apparent and true ileal digestibility (AID and TID) of protein and AAs from casein were measured.

**Results:**

The average flow of protein was 20.8 g/kg of dry matter intake (DMI). Basal protein loss, as estimated by regression, was 16.9 g/kg DMI. Glutamic acid, arginine, and aspartic acid (2.2, 1.4, and 1.2 g/kg DMI, respectively) were the AAs for which greater losses were seen. The AID of protein and AAs increased as the protein level in the diet increased. A higher increment in AID was observed between diets with 80 and160 g CP/kg of feed; this finding was mainly attributable to increases in glycine and arginine (46.1% and 18%, respectively). The TID of protein was 97.8, and the TID of AAs varied from 93.9 for histidine to 100.2 for phenylalanine.

**Conclusions:**

The basal endogenous protein loss in piglets was 16.9 g/kg DMI. Endogenous protein was rich in glutamic acid, aspartic acid, and arginine, which represented 32.7% of endogenous protein loss in weaning piglets. The TID of casein was high and varied from 93.0 for histidine to 100.2 for phenylalanine.

## Background

Many studies have investigated endogenous loss of proteins and amino acids (AAs) at the ileal level in growing pigs [1-3]. However, only a few studies have researched this subject in piglets [4-7]. Mariscal-Landín and Reis de Souza [6] reported high proline endogenous losses following prolonged intake (15 d) of a low protein diet in piglets, and similar effects of low protein or protein-free diets have been reported in growing pigs [1,8,9]. Further knowledge regarding AA ileal digestibility in piglets would be helpful during the formulation of diets for weaning piglets, rather than just using coefficients obtained from data for optimal diets in growing pigs, especially for cereals and vegetable proteins [10,11]. In order to achieve this goal, it is essential to determine the endogenous loss of protein and AAs in the ileum of piglets.

Therefore, the objectives of this study were: 1) measure endogenous ileal loss of protein and AAs in weaned piglets, avoiding changes in the AA profile of endogenous protein through prolonged intake of a low protein diet; 2) measure the apparent and true ileal digestibility (AID and TID, respectively) of protein and AAs derived from casein.

## Materials and methods

The experiment was performed at the experimental farm of CENID-Physiology. The care of animals used in this study was in accordance with the guidelines issued by the Mexican Official Standard for the Production, Protection and Use of Lab Animals [12] and the guidelines of the International Guiding Principles for Biomedical Research Involving Animals [13].

### Animals

Eighteen piglets (Duroc × Landrace; 6 per group), weaned at 18 ± 1 d of age and weighing 6 ± 1 kg, were used. The piglets were placed in individual metabolism cages provided with a self-feeder and a low pressure drinking nipple, in a temperature controlled room (26 ± 3°C). In order to modify the enzymatic profile of suckling animals as little as possible; piglets were fed from 18 to 20 d of age with a mixture of dry whole milk (80%) and maize starch (20%) 3 times/d, at 0800, 1300 and 1800 h, at a concentration of 30 g/kg body weight (BW). When the piglets were 21 d old, a T cannula was fit at the terminal ileum as previously described [14]. After surgery, a therapeutic treatment (penicillin 600,000 IU, streptomycin 750 mg, oxytetracycline 500 mg) was administered for 3 d, and the piglets received a casein diet containing 240 g of crude protein (CP)/kg for 8 d.

### Experimental diets

From the ninth d after surgery, piglets received 1 of 3 experimental treatments (Table 1). Casein was used as the sole protein source to provide 80, 160 or 240 g of CP/kg of feed. Because the low protein diets used in this study (80 or 160 g CP/kg) did not provide sufficient levels of lysine, methionine, threonine, or tryptophan, these AAs were supplemented to fulfill NRC nutrient requirements (Table 2) [15]. All 3 experimental diets contained the same amount of lactose and maize oil. To adjust calcium and phosphorus levels, calcium carbonate and dicalcium phosphate were used. Salt and vitamin and mineral premixes were included at levels that met or exceeded the NRC nutritional requirements [15]. Chromic oxide was added at a rate of 3 g/kg of feed as an inert marker.

**Table 1:** **Experimental diets composition (g/kg feed**^**1**^**)**

**g CP/kg feed**	**80**	**160**	**240**
Maize starch	677.3	604.2	523.3
Casein	89.8	180.0	270.1
Lactose	126.3	126.3	126.3
Maize oil	40.0	40.0	40.0
Calcium Phosphate			
(dicalcium)	32.1	27.9	23.8
Calcium carbonate		1.1	2.1
Salt	6.4	6.4	6.4
Vitamins^2^	3.6	3.6	3.6
Minerals^3^	1.2	1.2	1.2
Antioxidant^4^	0.2	0.2	0.2
Chromic oxide	3.0	3.0	3.0
Lysine HCl	1.7		
Tryptosine^5^	9.1	2.7	
DL Methionine	4.5	1.9	
L Threonine	4.8	1.5	
Protein, g/kg^6^	98.0	151.3	231.5
ED, MJ · kg^7^	15.1	15.1	15.1

^1^ As fed basis.

^2^ Vitamin premix that provided per kilogram of piglet diet: Vitamin A 10,200 IU, D 1,980 IU, E 60 IU, K 1.20 mg, Choline 967 mg, Niacin 36 mg, Pantothenic acid 17 mg, Riboflavin 7.2 mg, Vitamin B_12_ 40 μg, Thiamine 0.30 mg, Pyridoxine 0.31 mg, Biotin 0.08 mg, Folic acid 0.75 mg.

^3^ Mineral premix that provided per kilogram of piglet diet: Cu 14.4 mg, I 0.96 mg, Fe 120 mg, Mn 36 mg, Se 0.3 mg, Zn 144 mg.

^4^ ANTI-OX-HP, Compagnie Chimique d’Aquitaine.

^5^ 15/70 Tryptophan/Lysine Blend. Produced by Indukern de México.

^6^ Analyzed levels.

^7^ Estimated from INRA tables, [16].

**Table 2:** Protein and amino acids composition of experimental diets (g/kg DM)

**g CP/kg feed**	**80**	**160**	**240**
Protein	105.0	162.0	247.0
Essential amino acids			
Arginine	3.4	6.2	10.2
Histidine	2.6	4.9	7.8
Lysine	13.8	14.2	19.8
Isoleucine	4.6	8.6	14.1
Leucine	8.2	15.3	24.9
Valine	5.8	10.9	17.7
Methionine	7.0	7.5	6.9
Phenylalanine	4.7	8.6	15.1
Threonine	9.8	10.8	12.0
Non essential amino acids			
Alanine	2.8	5.2	8.4
Aspartic acid	6.2	11.5	18.9
Cystine	0.6	0.9	1.4
Glutamic acid	20.0	37.7	62.1
Glycine	1.8	3.4	5.3
Proline	8.2	17.3	23.7
Serine	4.8	9.0	14.8
Tyrosine	4.4	8.1	14.2

### Sample collection

Experimental diets were supplied starting on d 9 after surgery, according to schedules described previously. The experimental period lasted for 7 d, that is 5 d for adaptation and 2 d for collection of ileal digesta. Feed intake was fixed at 26 g/kg BW during the experimental period to maintain feed intake at a constant level in relation to BW for all animals. The piglets always had free access to water.

Ileal digesta were collected in plastic bags (5 cm × 3 cm) containing 2 mL of a 0.2 mol/L solution of HCl to minimize further bacterial activity. Bags were attached to the barrel of the cannula with a rubber band. Ileal digesta were collected continuously over the course of 12 h when the piglets were 36 and 37 d of age (d 16 and 17 after surgery). When ileal digesta filled the bags, they were transferred to a container and frozen at -20°C until lyophilization.

### Preparation of samples and chemical analysis

Ileal digesta were lyiophilized and ground in a laboratory mill by using a 0.5-mm mesh (Arthur H. Thomas Co. Philadelphia, PA). Experimental diets and ileal digesta were analyzed for dry matter (DM) and CP, according to AOAC methods 934.01 and 976.05 [17] respectively, and for chromic oxide according to the methods reported by Fenton and Fenton [18]. Sample preparation for AA analysis was performed following method 994.12 of AOAC [17], samples were hydrolysed in 6 mol/L HCl at 110°C for 24 h. Methionine and cystine were oxidized with performic acid before analysis [17]. Amino acids analysis was performed according to the method reported by Henderson et al. [19]. Tryptophan was not analyzed.

### Data analysis

Endogenous protein and AA losses in the ileal digesta were estimated using the following equation [20]:

$$\mathrm{ILAA}=\mathrm{AF}\;\times \;\left(\mathrm{ID}/\mathrm{IF}\right)$$

Where *ILAA* is the ileal loss of CP or AA expressed in g/kg DMI, *AF* is the nutrient concentration in ileal digesta (g/kg DM), *ID* is the marker concentration in the diet (g/kg DM), and *IF* is the marker concentration in the ileal digesta (g/kg DM).

The AIDs of DM, protein and AAs were estimated using the following equation [20]:

$$\mathrm{AID}=\;\left[1-\left[\left(\mathrm{ID}\;\times \;\mathrm{AF}\right)/\left(\mathrm{AD}\;\times \;\mathrm{IF}\right)\right]\right]\;\times \;100$$

Where *AID* is the apparent ileal digestibility of a nutrient in the diet, *ID* is the marker concentration in the diet (g/kg DM), *AF* is the nutrient concentration in the ileal digesta (g/kg DM), *AD* is the nutrient concentration in diet (g/kg DM), and *IF* is the marker concentration in the ileal digesta (g/kg DM). The calculi excluded crystalline amino acids (L-Lys-HCl, DL-Met, L-Thr and L-Trp) because they were considered completely digested at the end of the ileum [21,22].

Basal protein and AA ileal losses and the TIDs of protein and AAs from casein were estimated by linear regression [2]:

y=a+bx

Where *y* is the protein or amino acid amount disappearing from the digestive tract in terms of g/kg DMI, *a* is the protein or amino acid basal ileal loss in terms of g/kg DMI, *b* is the protein or amino acid disappearing in the digestive tract, (equivalent to the TID), and *x* is the intake in g/kg DMI. Regression was performed using the REG procedure of the SAS software [23]. Performing regression analysis in this way allowed us to obtain a high value of the regression coefficient *R*^*2*^, because almost the total amount of nutrient ingested disappeared at the ileal level. The intercept was negative because an endogenous loss was, by definition, an amount found at zero intake, and consequently, at zero intake there was no dietary nutrient absorption, only excretion. Nevertheless, the absolute value of the intercept using excreted (non-digestible) or absorbed (digestible) nutrient was the same and could be used as reported previously [2].

### Statistical analysis

Homogeneity of variance for all data was tested by Levene’s test using the hovtest of the SAS software [23]. Protein and AA Ileal losses and AID data were analyzed as a completely randomized design under the general model:

Yij=μ+Τi+eij

Where *Y*_*ij*_ is the dependent variable; *μ* is the general mean; *T*_*i*_ is the treatment_i_ 1, 2, 3; and *e*_*ij*_ is the experimental error, calculated using the GLM procedure of the SAS software [23]. The piglets were the experimental units for all analyses. Treatment means were compared using the Duncan method, an *α-*value of 0.05 was used to assess significance and orthogonal polynomial contrast were performed to find a linear or quadratic response [24].

## Results

### Protein and amino acids flow at ileal level

Protein flow was higher in the digesta of piglets fed 160 g CP/kg of feed than in the digesta of piglets fed 80 g CP/kg of feed (*P* < 0.05, Table 3). Moreover, significant differences among these groups were observed for the AAs valine (*P* < 0.05), threonine (*P* < 0.05), aspartic acid (*P* < 0.05), glutamic acid (*P* < 0.05), serine (*P* < 0.01) and histidine (*P* < 0.001). Protein flow was similar between digesta of piglets fed 160 or 240 g CP/kg of feed (*P* > 0.05).

**Table 3:** Protein and amino acids flow at the ileum level, in terms of dry matter intake (g/kg)

**g CP/kg feed**	**80**	**160**	**240**	**SEM**^**1**^	***P *** **value**^**2**^
Protein^C^	17.67^a^	22.10^b^	22.72^b^	0.77	0.05
Essential amino acids					
Arginine	1.25	1.56	1.47	0.06	NS
Histidine^B^	0.36°	0.52^b^	0.74^b^	0.04	0.001
Lysine	0.61	0.74	0.71	0.04	NS
Isoleucine^B^	0.65°	0.88^b^	0.93^b^	0.03	0.01
Leucine^E^	0.91	1.17	1.11	0.04	NS
Valine^B^	0.81°	1.08^b^	1.11^b^	0.04	0.05
Methionine	0.30	0.31	0.25	0.02	NS
Phenylalanine^E^	0.55	0.67	0.60	0.03	NS
Threonine^B^	1.01°	1.34^b^	1.43^b^	0.05	0.05
Non essential amino acids					
Alanine^D^	0.97	1.24	1.22	0.05	NS
Aspartic acid^C^	1.26°	1.77^b^	1.73^b^	0.08	0.05
Cystine	0.32	0.37	0.39	0.05	NS
Glutamic acid^C^	2.33°	3.65^b^	3.76^b^	0.21	0.05
Glycine	0.94	0.97	1.02	0.04	NS
Proline^D^	1.03	1.37	1.40	0.08	NS
Serine^A^	1.04°	1.49^b^	1.69^b^	0.06	0.01
Tyrosine^E^	0.49	0.61	0.55	0.03	NS

^1^ Standard error of the mean.

^2ab^ Means with different superscript differ at probability level show in the fifth column.

*NS* non significant.

^A^ Linear effect (*P* < 0.001), ^B^ Linear effect (*P* < 0.01), ^C^ Linear effect (*P* < 0.05), ^D^ Linear effect (*P* < 0.10), ^E^ Quadratic effect (*P* < 0.10).

For all 3 groups, the average protein flow was 20.8 g/kg DMI. Protein was rich in glutamic acid, aspartic acid, arginine and threonine (3.2; 1.6; 1.4 and 1.3 g/kg DMI, respectively). These amounts represented 17.6%, 8.7%, 7.9% and 7.0% of total AAs. AAs with lower flow included methionine, histidine and tyrosine (0.3, 0.5, and 0.5 g/kg DMI, respectively); these flow represented 1.6%, 3.0%, and 3.1% of total AA flow, respectively.

### Basal endogenous losses

The basal endogenous protein ileal loss estimated by regression was 16.9 g/kg DMI (Table 4). Glutamic acid, arginine and aspartic acid were the AAs exhibiting larger basal endogenous ileal losses (2.2, 1.4, and 1.2 g/kg DMI, respectively). AAs with lower endogenous losses included histidine, methionine, and tyrosine (0.2, 0.4 and 0.5 g/kg DMI, respectively). All regressions were significant (*P* < 0.001) and had determination coefficients of at least 0.98.

**Table 4:** **Basal ileal losses (g/kg DMI) and true ileal digestibility estimated by regression**^**1**^

	**BEL**^**2**^		**TID**^**3**^		***R***^***2*** **(5)**^
	***a***	**S.D.**^**4**^	***b***	**S.D.**	
Protein	16.95	3.69	97.8	0.3	0.99
Essential amino acids					
Arginine	1.36	0.26	99.0	3.8	0.98
Histidine	0.18	0.10	93.0	1.9	0.99
Lysine	0.60	0.16	99.4	1.1	0.99
Isoleucine	0.64	0.17	98.0	1.8	0.99
Leucine	0.98	0.19	99.5	1.1	0.99
Valine	0.81	0.18	98.4	1.5	0.99
Methionine	0.35	0.09	97.0	9.0	0.99
Phenylalanine	0.63	0.10	100.2	1.0	0.99
Threonine	0.96	0.21	96.0	2.6	0.99
Non essential amino acids					
Alanine	0.99	0.20	97.2	3.5	0.98
Aspartic acid	1.25	0.26	97.1	2.1	0.99
Cystine	0.30	0.07	95.3	4.8	0.96
Glutamic acid	2.18	0.69	97.1	1.7	0.99
Glycine	0.95	0.13	99.3	3.4	0.98
Proline	0.97	0.27	98.1	1.6	0.99
Serine	0.91	0.26	95.0	2.6	0.99
Tyrosine	0.55	0.09	100.1	1.0	0.99

^1^ According to equation: ***Y = a + bX***.

^2^*BEL* Basal endogenous losses (***a*** = intercept, protein or amino acids endogenous losses at ileal level).

^3^*TID* True ileal digestibility (***b*** = slope, proportion of protein or amino acid that disappeared in the digestive tract, per protein or amino acid unit intake).

^4^*SD* Standard deviation of the intercept ***(a)*** or slope ***(b)***.

^5^***R***^***2***^ Coefficient of determination for regression straight line.

### AID

The AIDs of protein, arginine, leucine, isoleucine, valine, phenylalanine, methionine, aspartic acid, serine, glycine, alanine and tyrosine, increased as casein levels increased (Table 5). The AIDs of histidine, cystine and proline differed significantly between piglets fed 80 and 160 g CP/kg feed, while the AIDs of these AAs were similar between piglets fed 160 and 240 g CP/kg of feed. The AID of lysine was different between piglets consuming 80 and 240 g CP/kg, and the AID observed in piglets fed with 160 g CP/kg was similar to those of the other 2 treatments. The AID of glutamic acid was similar in piglets consuming 80 and 160 g CP/kg and lower than that in piglets consuming 240 g CP/kg. However, all amino acids and protein showed a linear increment (*P <* 0.01) in their AIDs as protein level increased in the diet.

**Table 5:** Apparent ileal digestibility

**g CP/kg feed**	**80**	**160**	**240**	**SEM**^**1**^	***P *** **value**^**2**^
Protein^A^	82.8°	86.5^b^	90.4^c^	0.06	0.001
Essential amino acids					
Arginine^A^	62.3°	73.5^b^	84.1^c^	1.17	0.001
Histidine^A^	85.5°	89.0^b^	89.8^b^	0.71	0.05
Lysine^A^	93.4°	94.5^ab^	96.1^b^	0.35	0.05
Isoleucine^A^	85.6°	89.3^b^	92.6^c^	0.39	0.001
Leucine^A^	88.7°	92.0^b^	95.1^c^	0.35	0.001
Valine^A^	85.7°	90.7^b^	93.1^c^	0.39	0.001
Methionine^A^	89.7^a^	93.3^b^	96.9^c^	0.42	0.001
Phenylalanine^A^	88.0°	92.0^b^	95.6^c^	0.38	0.001
Threonine^A^	76.7°	80.2^b^	86.9^b^	0.78	0.001
Non essential amino acids					
Alanine^A^	65.5°	74.9^b^	84.0^c^	1.09	0.001
Aspartic acid^A^	79.5°	83.9^b^	90.0^c^	0.72	0.001
Cystine^A^	51.4^a^	72.3^b^	79.5^b^	4.26	0.01
Glutamic acid^A^	88.1°	89.8^a^	93.4^b^	0.55	0.01
Glycine^A^	47.7^a^	69.7^b^	79.3^c^	1.23	0.001
Proline^A^	87.1^a^	91.7^b^	93.6^b^	0.49	0.001
Serine^A^	78.1°	82.5^b^	87.5^c^	0.73	0.001
Tyrosine^A^	88.8^a^	92.1^b^	95.7^c^	0.37	0.001

^1^ Standard Error of the Mean.

^2abc^ Means with different superscript differ at probability level show in the fifth column.

^A^ Linear effect (*P* < 0.001).

The AAs with the greatest differences in their AIDs between piglets consuming 80 and 160 g CP/kg were glycine (46.1%), arginine (18%), valine (16.3%) and alanine (14.4%).

The difference in the AID of protein between piglets consuming 80 and 240 g CP/kg feed was 9.2%, which was much lower differences in the AIDs of glycine (66.2%), arginine (35%) and alanine (28.2%) between these groups. The lowest difference in AID between these groups was for Lysine (2.9%).

### TID

The TID of protein was 97.8, and the average TID for AA was 97.6. The minimum TID value was obtained for histidine (93.0) and the maximum TID value was obtained for phenylalanine (100.2). The TID values for lysine and threonine were 99.4 and 96.0, respectively (Table 4).

## Discussion

In this study, we estimated endogenous protein and AA ileal losses in piglets. Our data demonstrated that endogenous protein was rich in glutamic acid, aspartic acid, and arginine, which represented 32.7% of endogenous protein loss in weaning piglets. Moreover, the TID of casein was high, varying from 93.0 for histidine to 100.2 for phenylalanine.

### Ileal losses

The protein and AA ileal losses found in this study were similar to those reported by Mariscal-Landín and Reis de Souza [6], except that proline losses were higher in the earlier study. This difference was likely to be attributable to the fact that we used higher dietary protein levels and that lysine, methionine, threonine and tryptophan were supplemented to fulfill NRC requirements in the current study [15]. Another reason could be the duration of the experimental period: 7 d in the current study compared to 14 d in the previous study. This shorter period was used to avoid increased proline loss caused by prolonged consumption of a low protein diet [9].

AA supplementation and the short period of consumption of the low protein diet could reduce muscle catabolism, thereby decreasing blood glutamine levels. These effects could lead to suppression of proline synthesis because enterocytes use glutamine as proline precursor [25-27]. Moreover, proline synthesis dependent of pyrroline-5-caboxylate, has been associated with increased proline loss in growing pigs fed protein-free diets [1,8,28]. Because the activity of pyrroline-5-carboxylate reductase, the enzyme responsible for proline synthesis, is greater than the activity of proline oxidase, the enzyme responsible for proline degradation [1].

The arginine losses reported here agree with the results of some previously reported studies Leibholz [29] and Mariscal-Landín and Reis de Souza [6], but disagree with the results of studies by Eklund [4,5]. These differences are likely due to differences in study design; Eklund used during 4 periods of 7 d each with the same piglets and reported values as the mean of the 4 periods. Our values represented only those obtained during the fourth wk of age, which was also observed in studies by Leibholz [29] and Mariscal-Landin and Reis de Souza [6].

Additionally, the glycine losses reported in our study agree with values reported in 4-wk-old piglets fed a protein-free diet [30] and in 4- and 5-wk-old piglets [6]. Glycine losses are associated with biliary secretion because glycine represents 95% of amino nitrogen present in biliary salts [31].

### Ileal digestibility

The AID for protein from casein (86.6 on an average) and for AA were similar to those reported by Mariscal-Landín and Reis de Souza [6]. Additionally, the AIDs for glycine, arginine and alanine (47.7, 62.3 and 65.5 respectively) were the most affected by protein levels. Glycine has been reported as the least digestible AA from casein [6,32], as observed in this study, because lacteal proteins, which include casein, have a low glycine content, and glycine endogenous losses are important [30]. Both factors interact to reduce the AID of glycine. Moreover a combination of the same factors could explain the low AID of arginine because lacteal proteins have low arginine content [33] and because endogenous protein is rich in arginine [6,29].

The lack of variation in the AIDs of lysine, histidine, threonine, cystine and proline between treatments (160 and 240 g CP/kg of feed) could be due to the high levels of these AAs in protein. Jørgensen [34] fed growing pigs a casein-based diet (13%, 16%, 19%, and 22% CP) and observed differences between the AIDs of AAs only for pigs fed 13% and 16% CP. No differences were observed in groups fed 16%, 19% or 22% CP. The high TID values (greater than 93.0) were due to high casein digestibility and its low induction of endogenous secretion [35]; because of these characteristics, casein is often used to measure endogenous losses. In our study, the TIDs of AAs were similar to those reported previously [4,6].

## Conclusions

The basal endogenous protein loss in piglets was 16.9 g/kg DMI; Endogenous protein was rich in glutamic acid, aspartic acid and arginine, which represented 32.7% of endogenous protein loss in weaning piglets. The TID of casein was high and varied from 93.0 for histidine to 100.2 for phenylalanine.

## Abbreviations

AA: Amino acids; AID: Apparent ileal digestibility; TID: True ileal digestibility; BW: Body weight; DM: Dry matter; CP: Crude protein.

## Competing interests

The authors declare that they have no competing interests.

## Authors’ contributions

All authors contributed equally to this research. All authors read and approved the final manuscript.
